# 16 years of gelatinous zooplankton seasonal abundances reveal food availability and temperature as key drivers in Arctic latitudes

**DOI:** 10.1038/s41598-026-60064-1

**Published:** 2026-06-30

**Authors:** Christian W. G. Detsch, Thomas Juul-Pedersen, Gerlien Verhaegen, Charlotte Havermans, Doreen Kohlbach

**Affiliations:** 1 University of Bremen, James-Watt-Straße 1, 28359 Bremen, Germany; 2 Alfred Wegener Institute Helmholtz-Center for Polar and Marine Research, Am Handelshafen 12, 27570 Bremerhaven, Germany; 3 Greenland Institute of Natural Resources, Kivioq 2, 3900 Nuuk, Greenland; 4 Advanced Institute for Marine Ecosystem Change (WPI-AIMEC), Japan Agency for Marine-Earth Science and Technology (JAMSTEC), 3173-25 Showa-machi, Kanazawa-ku, Yokohama-shi, Kanagawa 236-0001 Japan

**Keywords:** Jellyfish, Abundances, Environmental drivers, Greenland Ecosystem Monitoring (GEM), Long-term monitoring, Climate change, Ecology, Ecology, Ocean sciences

## Abstract

Year-round long-term monitoring programs are essential for assessing ecosystem functioning and change. In the Arctic, however, continuous multi-year sampling covering all seasons remains scarce. Using monthly observations from the Greenland Ecosystem Monitoring program (2005–2021) in Nuup Kangerlua (SW Greenland), collected with triplicate 0–100 m net hauls and concurrent environmental profiling, we characterized seasonal and multiannual abundance patterns of major gelatinous zooplankton (GZP) groups (Ctenophora, Appendicularia, Cnidaria, and Chaetognatha) and assessed their responses to temperature and salinity, as well as food availability (i.e., chlorophyll *a*, biomass of small copepods, small *Calanus* spp. (CI–CIII), large *Calanus* spp. (CIV–CVI) and copepod nauplii abundance), the latter being drivers rarely included in long-term studies. All GZP groups showed strong seasonal variability in abundances, with peak abundances in summer. Hereby, copepod availability showed correlations with GZP abundance that were equal to or stronger than those of temperature. Chlorophyll *a* correlated weakly with GZP abundance directly, but remained a central driving factor of prey availability and therefore still indirectly influenced carnivorous GZP dynamics. Salinity showed mostly negative associations. Indicator species analyses identified *Oikopleura* spp., *Parasagitta* spp., *Aglantha digitale*, and undetermined Hydromedusae as characteristic of warm, food-rich summer conditions. Additionally, undetermined Chaetognatha were an indicator taxon for the entire productive season. These findings demonstrate that resolving Arctic change requires year-round observations that integrate trophic context, as food availability can rival or exceed physical drivers in structuring GZP communities, as demonstrated here with copepods and primary production.

**Supplementary Information:**

The online version contains supplementary material available at 10.1038/s41598-026-60064-1.

## Introduction

The Arctic is one of the fastest changing regions with respect to climate change^1^. These changes induce poleward distribution shifts of boreal and boreal–Arctic species, associated with warmer temperatures and increased influence of Atlantic water masses^2^. Long-term monitoring programs are essential to document these shifts, place them into ecological context, and support predictions of future ecosystem trajectories. In the pelagic realm, shifts from a lipid-rich Arctic zooplankton community to one composed of smaller boreal individuals have been reported all across high latitude systems^3–17^. These shifts happening at the base of many trophic pathways may have far-reaching implications for the entire ecosystem^2,18,19^. Despite the importance in understanding Arctic marine functioning, community shifts and the effects of environmental change, long-term observations of the pelagic ecosystem are still rare and only few are established, for example in the Canadian, European and Russian Arctic^17,20–22^. Yet, these monitoring programs often lack methodological continuity, fixed geographical locations, high temporal resolution, especially during winter when harsh environmental conditions severely limit year-round monitoring in high latitudes^22^, or duration over decades. In particular, this lack of year-round coverage constrains our ability to assess seasonal ecosystem functioning and its link to environmental drivers, particularly for strongly seasonal but ecologically important groups such as gelatinous zooplankton.

Gelatinous zooplankton (GZP) comprise representatives from the taxonomic groups Ctenophora, Appendicularia, Cnidaria, and sometimes also Chaetognatha, often considered as a “semi-gelatinous” group (i.e., with dry weight > 5% wet weight)^23^. They are increasingly recognized for their ecological importance, also in high-latitude regions, with rising GZP occurrences now widely reported for different sub-Arctic and Arctic ecosystems^24–28^. Since many GZP species that are abundant in the Arctic have a boreal-arctic distribution, occurrences of Ctenophora and Cnidaria in the northeast Norwegian Sea and Cnidaria in the southwest Barents Sea were significantly positively correlated with sea surface temperature^28^. A similar association of high abundances with warm temperatures was made for large scyphozoans in the Barents Sea^26^. These observations mostly rely on abiotic variables, tending to overlook ecological drivers like food availability and predation pressure. In the same line, a general poleward increase in suitable habitat until the end of the century due to retreating sea ice and rising water temperature has been projected for abundant GZP species in high-latitude systems including *Aglantha digitale* (Cnidaria), *Periphylla periphylla* (Cnidaria), *Cyanea capillata* (Cnidaria), *Oikopleura vanhoeffeni* (Appendicularia), *Fritillaria borealis* (Appendicularia), *Mertensia ovum* (Ctenophora), and *Beroe* spp. (Ctenophora)^29^. Even though current observations of increasing GZP populations are often explained by warming seawater temperatures, the consideration of different ecological factors such as changing trophodynamics should also be considered^24,28,30–32^.

The ecological importance of GZP has gained increasing attention in recent years. Traditionally considered trophic dead ends, they are now recognized as important grazers, predators and prey. As planktivorous predators, carnivorous GZP can exert strong top-down control, with some species capable of drastically reducing stocks of fish larvae and copepods^25,26,33,34^. While the traditional view of (carnivorous) GZP as typical predators on small crustaceans and fish larvae remains unquestioned^35,36^, their importance as prey for higher trophic levels often appears more ambiguous and underestimated^37^. Nowadays, their role as prey becomes increasingly better understood^38–42^. The common hydromedusa *A. digitale* is one of the most widespread hydrozoans in the Arctic and adjacent waters. Its carbon and nitrogen content are comparable to that of ten small copepods such as *Pseudocalanus* spp.^43^, highlighting its nutritional value. In high-latitude systems, GZP is consumed by amphipods and shrimps^38,39,41^, while fish regularly feed on Cnidaria and Chaetognatha alongside Crustacea^40,44^. Further, Appendicularia and Chaetognatha serve as important food sources for several copepod species^45,46^ as well as for immature polar cod^47^. In addition, Chaetognatha are also preyed upon by Cephalopoda^48,49^ and seabirds^50,51^, underscoring their diverse and complex role in (sub-)Arctic food webs.

The accurate assessment of GZP can be challenging. Due to the fragile body structure, GZP retrieved with traditional plankton-net sampling is often damaged, preventing accurate species-level identification. Thus, biodiversity monitoring efforts for GZP remain scarce and often rely on expert knowledge or molecular tools^52^. Furthermore, most GZP data derives from the summer period, particularly in high latitudes, despite their expected increase in abundance and ecological relevance. Summer sampling captures many taxa in their adult pelagic stages (e.g., medusae), but their full seasonal life cycles are often more complex, involving active or resting pelagic phases and, in some species, benthic overwintering stages^53^. Several Cnidaria and Ctenophora overwinter as adults, though strategies vary: large medusae such as *Chrysaora melanaster* overwinter near the seafloor, feeding on benthic fauna, whereas smaller hydromedusae (e.g., *A. digitale*) and ctenophores (e.g., *M. ovum*) remain in the epipelagic zone^53^. Chaetognaths also employ distinct overwintering strategies while remaining pelagic; *Parasagitta elegans* enters a low-feeding resting stage, whereas *Eukrohnia hamata* likely relies on wax ester reserves during winter^54,55^. Appendicularians appear to remain active year-round^56^. Thus, in order to understand dynamics in GZP populations, reliable multiyear baselines of their abundances and ecological drivers throughout the entire year are essential, especially since GZP abundances can exhibit strong fluctuations^57^, due to seasonal variability in vertical distribution^58^, advection^59^, life-cycles^53^ or environmental conditions^29^. For the assessment of ecological drivers of these dynamics, records on prey availability support data of temperature, salinity, and chlorophyll *a* concentration. While pico- and nanoplankton concentrations are mostly influencing grazing Appendicularia populations directly, the effects on Ctenophora, Cnidaria, and Chaetognatha are rather indirect through their herbivorous prey (e.g., copepods). Hence, the Greenland Ecosystem Monitoring (GEM) program established a high-resolution monthly time series in Nuup Kangerlua at the outer fjord in southwest Greenland assessing abiotic and biotic environmental changes, including mesozooplankton dynamics, collected with triplicate 0–100 m WP2 net hauls alongside CTD-derived hydrography and chlorophyll *a* measurements. Using this high-resolution temporal dataset (October 2005–August 2021), we aimed to characterize the seasonal and multiannual dynamics of Ctenophora, Appendicularia, Cnidaria, and Chaetognatha, providing a baseline for future ecological assessments of GZP and identify environmental drivers of their seasonal abundance patterns. We predicted water temperature to be a strong driver of the observed seasonal dynamics and hypothesized a stronger coupling of high GZP abundances to prey phenology than to phytoplankton concentration due to the primarily carnivorous diet of most GZP taxa, except for Appendicularia. Therefore, we expected high GZP abundances during summer, a period often associated with warm temperatures and elevated food availability that can favor GZP population growth^29,33,53^. Addressing these objectives will help to observe, evaluate and predict GZP of high latitude marine systems in future research efforts.

## Results

### Seasonal hydrography patterns

**Fig. 1:**
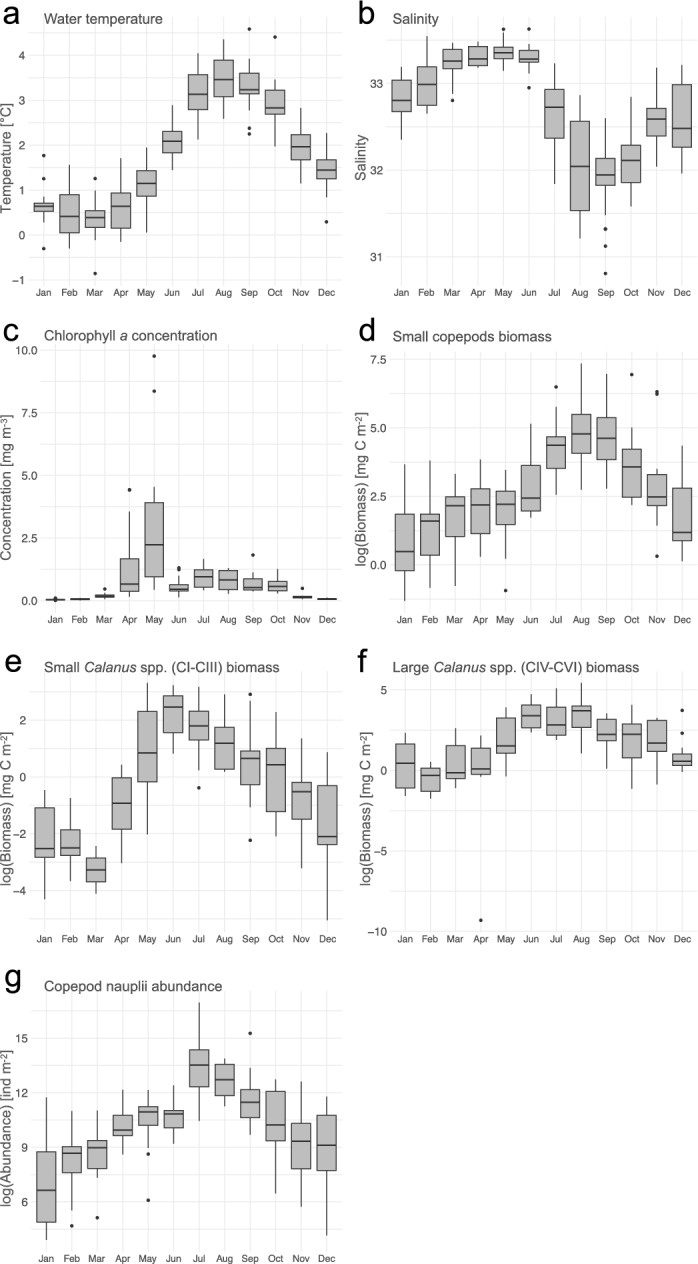
Environmental variables from the surface (mean 0–100 m) at GF3 in Nuup Kangerlua from October 2005–August 2021 on a monthly scale including water temperature (**a**), salinity (**b**), chlorophyll *a* concentration (**c**), log-transformed small copepod biomass (**d**), log-transformed small *Calanus* spp. biomass (**e**), log-transformed large *Calanus* spp. biomass (**f**), and log-transformed copepod nauplii abundance (**g**) at GF3 in Nuup Kangerlua from October 2005–August 2021.

Environmental parameters showed pronounced seasonal variation throughout the study period (Fig. 1). Mean water temperature increased from winter minima at 0.4 ± 0.5 °C in March to summer maxima of 3.5 ± 0.5 °C in August, reflecting the typical annual thermal cycle. Salinity exhibited an opposite trend, with slightly higher values during late winter and spring (up to 33.4 ± 0.1) and a gradual decrease towards late summer and early autumn (~ 31.9 ± 0.4), due to enhanced freshwater influence. Chlorophyll *a* concentrations indicated a marked spring bloom, with mean values rising from < 0.1 µg m⁻3 in winter to 3.0 ± 2.7 µg m⁻3 in May, followed by moderate levels through summer and autumn.

### Seasonal copepod community patterns

Copepod groups likewise exhibited pronounced seasonal structure (Fig. 1). Mean small copepod biomass increased steadily from winter minima (6.3 ± 11.3 mg C m^−2^ in January) through spring (13.4 ± 8 mg C m^−2^ in April) to a marked summer maximum at 258.5 ± 414.2 mg C m^−2^ in August, before declining towards early-winter levels (14.6 ± 22.9 mg C m^−2^ in December). Small *Calanus* showed an even stronger seasonal contrast, remaining extremely low during winter and early spring (0.2 ± 0.2 to 0.05 ± 0.05 mg C m^−2^ from January to March), rising rapidly in late spring, and peaking in early summer (12.3 ± 8.4 mg C m^−2^ in June), followed by a gradual decrease through autumn. In contrast, large *Calanus* biomass increased earlier in the year, shifting from moderate winter values (~ 3 mg C m^−2^) to sustained high levels throughout summer and early autumn, with maxima in August of 58.3 ± 71.8 mg C m^−2^, and remaining comparatively elevated into October with 14.3 ± 17.2 mg C m^−2^. Copepod nauplii abundance displayed the greatest seasonal amplitude of all zooplankton variables, increasing from winter means (16 × 10^3^ ± 41 × 10^3^ ind m^-2^ in January) to a pronounced midsummer peak (3.3 × 10^6^ ± 7 × 10^6^ ind m^−2^ in July), and subsequently declining through autumn to late-year levels at 35 × 10^3^ ± 48 × 10^3^ ind m^−2^ in December. Collectively, these patterns indicate a tightly synchronized seasonal succession, with early increases in *Calanus*, followed by peaks in small copepods and nauplii during mid- to late summer.

Hereby of notable importance was the driving role of phytoplankton (chlorophyll *a*) on the copepod community. As this relationship might underlie a lag between consumption and production, the correlation between chlorophyll *a* and small copepod biomass, small *Calanus* spp. biomass, large *Calanus* spp. biomass and copepod nauplii abundance was investigated with Spearman rank correlation tests and a 0–4 months lag (Supplementary Fig. 1 & Supplementary Table 1). Small and large *Calanus* exhibited the strongest response overall, peaking near a 1-month lag (ρ ≈ 0.56, *p* < 0.001), suggesting rapid population growth following phytoplankton increases. Nauplii displayed moderate peak correlations (ρ ≈ 0.46, *p* < 0.001) at short lags (1–2 months). Small copepods showed a weaker but persistent positive relationship across lags, with maximum correlation around 1–2 months (ρ ≈ 0.44, *p* < 0.001). Exact correlations coefficients should be considered with care as they are based on regularized data.

### Overall GZP composition

**Fig. 2:**
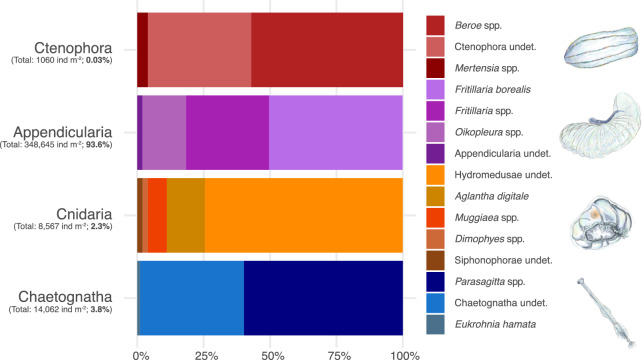
Relative contribution to the total abundance of the identified taxa to their respective gelatinous zooplankton group.

The GZP community retrieved from the upper 100 m at the outer fjord of Nuup Kangerlua from October 2005 to August 2021 consisted of four major groups: Appendicularia (93.6% of total GZP abundance), Chaetognatha (3.8%), Cnidaria (2.3%), and Ctenophora (0.03%), (Fig. 2 & Supplementary Table 2). Appendicularia, the most dominant group, was largely represented by the genus *Fritillaria,* which contributed over 80% to the overall group abundance. Specifically, *F. borealis* accounted for half of the total Appendicularia abundance (175,489 ind m^−2^; 50.3%) and *Fritillaria* spp. added another one third (108,852 ind m^−2^; 31.2%). *Oikopleura* spp. had a lower contribution of 16.5% (57,663 ind m^−2^) over the study period, while a minor portion of the Appendicularia community remained unidentified (6,641 ind m^−2^; 1.9%). Within Chaetognatha, *Parasagitta* spp. was the most abundant taxon with a total abundance of 5,747 ind m^−2^ (59.9%), and *Eukrohnia hamata* the least abundant (144 ind m^−2^; 1%). However, over one third of the Chaetognatha could not be identified (5,496 ind m^−2^; 39.1%). Cnidaria only comprised hydrozoan taxa as scyphozoans were not detected. The majority of these hydrozoans remained unidentified (6,383 ind m^−2^; 74.5%). *Aglantha digitale* accounted for 14.3% (1,228 ind m^−2^) of the cnidarian abundance. Siphonophores contributed the remaining proportion: *Muggiaea* spp. with 7.2% and 620 ind m^−2^, while *Dimophyes* spp. and unidentified siphonophores accounted each for 2% and 168 ind m^−2^. Ctenophora abundance was mostly dominated by 57.2% of *Beroe* spp., which accounted in total (all entries summed up) for 606 ind m^−2^. Unidentified Ctenophora contributed with 38.9% or 412 ind m^−2^ and *Mertensia* spp. with 4% or 42 ind m^-2^ to the total Ctenophora abundance.

### Seasonal GZP abundance patterns

**Fig. 3:**
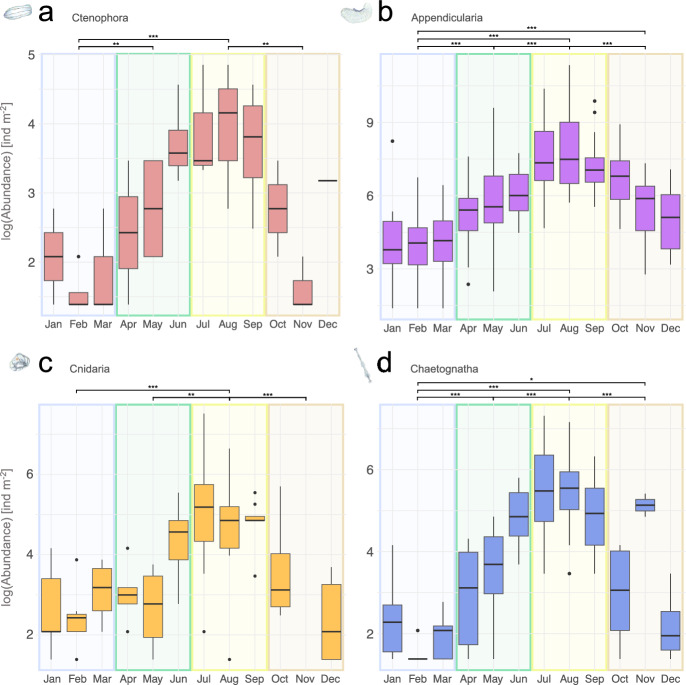
Seasonal dynamics of logarithmically transformed abundance of Ctenophora (**a**), Appendicularia (**b**), Cnidaria (**c**), and Chaetognatha (**d**) per month from October 2005–August 2021. Background shadings indicate seasons: winter (January–March), spring (April–June), summer (July–September), and autumn (October–December). Brackets indicate significant differences between seasons assessed with Tukey HSD tests (* = *p* ≤ 0.05; ** = *p* ≤ 0.01; *** = *p* ≤ 0.0001). Note the differing scale on the y-axes.

The four GZP groups exhibited pronounced seasonal variability in their abundances in the upper 100 m (Fig. 3). Statistical parameters of pairwise Tukey HSD tests are provided in Supplementary Table 3. All four GZP groups showed strong significant seasonal patterns (ANOVA, *p* ≤ 0.0001 for all). Ctenophora (number of samples *n* = 36) showed significantly higher abundances during spring and summer compared to winter and autumn. Mean abundance increased from 5 ± 2 ind m^−2^ in February to 69.3 ± 56.2 ind m^−2^ in August, then declined towards autumn (24–5.3 ± 2.3 ind m^−2^). The elevated value observed in December (24 ind m^−2^) likely reflects a sampling artefact due to the small sample size (*n* = 1). Appendicularia (*n* = 152) exhibited significantly greater abundances in summer compared to all other seasons. The population gradually increased over spring (mean: 433.1 ± 591.1–1422.3 ± 3602.6 ind m^−2^), peaked in August at 11,849.8 ± 24,363.8 ind m^−2^, and then declined markedly towards winter (301.9 ± 342.8 ind m^−2^). Cnidaria (*n* = 75) also had abundances significantly higher in summer than in winter and spring. After low densities from January to May (14.5 ± 13.9–27 ± 18.3 ind m^−2^), mean densities rose sharply in July (340.7 ± 539.6 ind m^−2^) and remained elevated until September (140 ± 63.9 ind m^−2^) before declining rapidly in autumn until 16.4 ± 16 ind m^−2^. Chaetognatha (*n* = 97) displayed pronounced seasonal variability with abundances significantly higher in spring and summer compared to winter and autumn. Mean densities increased from winter (4.6 ± 1.5 ind m^−2^) to peaks during July–August (up to 428.5 ± 453.9 ind m^−2^), before decreasing again in autumn (21.3 ± 31.6–7.3 ± 12.7 ind m^−2^). The relatively high abundance in November (176 ± 67.8 ind m^−2^) may be due to limited sampling (*n* = 2).

### Correlations with environmental drivers

**Fig. 4:**
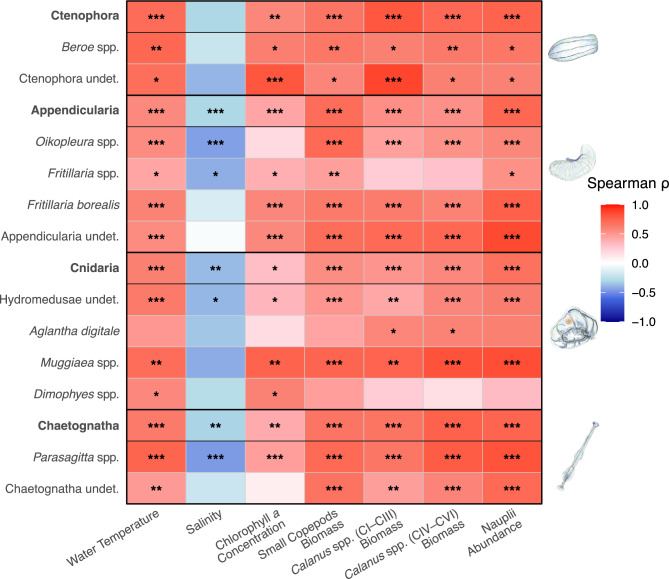
Spearman rank correlation heatmap showing pairwise relationships between the abundance of gelatinous zooplankton taxa (rows) and environmental drivers (columns: temperature, salinity, chlorophyll *a* concentration, small copepod biomass, small *Calanus* spp. biomass, large *Calanus* spp. biomass and copepod nauplii abundance) in the upper 100 m at Nuup Kangerlua between October 2005 and August 2021. Tile color indicates the correlation coefficient (ρ), scaled from –1 (blue) to + 1 (red). Significance levels are indicated by asterisks within tiles (* = *p* ≤ 0.05; ** = *p* ≤ 0.01; *** = *p* ≤ 0.0001).

Spearman rank correlations were used to examine relationships between GZP abundances and seven ecological variables: temperature, salinity, chlorophyll *a* concentration, small copepod biomass, small *Calanus* spp. biomass, large *Calanus* spp. biomass, and copepod nauplii abundance in the upper 100 m (Fig. 4; Supplementary Table 4). Hereby, the entire sampling period (October 2005–August 2021) was regarded, but taxa with less than 10 records were excluded to facilitate statistical robustness.

Ctenophora showed the strongest positive correlations of total abundance with both *Calanus* spp. variables, copepod nauplii abundance, and temperature (ρ = 0.7–0.8; *p* ≤ 0.0001).Weaker correlations were determined with chlorophyll *a* concentration (ρ = 0.57; *p* = 0.003), as well as small copepod biomass (ρ = 0.66; *p* ≤ 0.0001), while none were observed with salinity. *Beroe* spp. displayed similar positive correlations with water temperature and equal positive correlations with prey availability variables, while Ctenophora undet. showed very strong correlations to chlorophyll *a* concentration and the biomass of small *Calanus* spp. *Mertensia* spp. was excluded due to insufficient data.

Appendicularia abundance correlated the strongest positively with the biomass of small copepods and nauplii abundance (ρ ≈ 0.7; *p* ≤ 0.0001). Water temperature (ρ = 0.43–0.56; *p* ≤ 0.0001), chlorophyll *a* concentration and biomass of both *Calanus* groups correlated also positively but weaker, whereas salinity correlated negatively (ρ = − 0.3; *p* = 0.0009). *Fritillaria* spp. and *F. borealis* correlated positively with chlorophyll *a* concentration, whereas *Oikopleura* spp. showed a strong negative correlation with salinity.

Cnidaria exhibited positive relationships of total abundance with water temperature (ρ ≈ 0.6; *p* ≤ 0.0001) and nauplii abundance, a weaker positive correlation with the biomass of small copepods, small *Calanus* spp. and large *Calanus* spp. (ρ = 0.51–0.56; *p* ≤ 0.0001), and a very weakly with chlorophyll *a* concentration (ρ = 0.32; *p* = 0.02). A negative correlation of Cnidaria abundance was determined with salinity (ρ = − 0.38; *p* = 0.0043). Among taxa, the trachymedusa *A. digitale* correlated only with the biomass of *Calanus* spp. The siphonophores *Dimophyes* and *Muggiaea* showed contrasting correlations: while *Dimophyes* correlated only with water temperature and chlorophyll *a* concentration positively, *Muggiaea* correlated especially strong with large *Calanus* spp. and nauplii abundance. Siphonophorae undet. did not provide enough records (*n* < 10) to be included.

Chaetognatha abundance correlated positively with the biomass of small copepods, small *Calanus* spp., large *Calanus* spp., nauplii abundance, and water temperature (ρ = 0.63–0.75; *p* ≤ 0.0001), weaker with chlorophyll *a* concentration (ρ = 0.4; *p* = 0.001), but negatively with salinity (ρ = – 0.31; *p* = 0.0075). Similar patterns were observed for *Parasagitta* spp., whereas undetermined Chaetognatha correlated strongly only with small copepod biomass and nauplii abundance. *E. hamata*. was excluded due to too few records (*n* < 10).

### Multivariate analyses

**Fig. 5:**
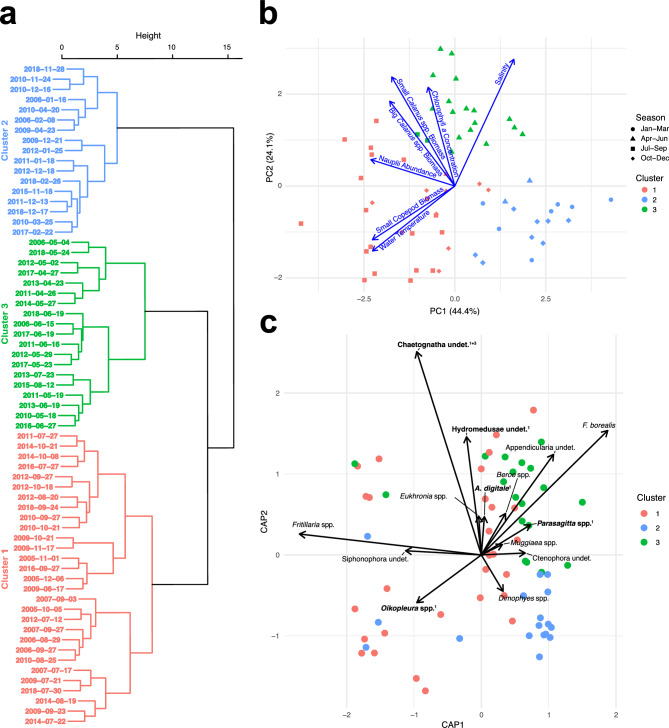
Multivariate analyses of environmental variability and taxa composition between October 2005 and August 2021 in the upper 100 m at Nuup Kangerlua. **(a)** Hierarchical clustering of sampling dates based on seven environmental variables (temperature, salinity, chlorophyll *a*, small copepod biomass, small *Calanus* spp. biomass, large *Calanus* spp. biomass, nauplii abundance) using Euclidean distance and Ward’s linkage. Colors indicate the three clusters (1 = red, 2 = blue, 3 = green). **(b)** Principal Component Analysis (PCA) ordination of samples based on the same variables. Axes show PC1 (44.4%) and PC2 (24.1%) explained variance. Colors denote clusters and symbol shapes indicate seasons. **(c)** Canonical Analysis of Principal Coordinates (CAP) ordination relating taxa composition to environmental gradients. Arrows represent fitted taxa vectors. Indicator taxa are shown in bold, with superscript numbers indicating associated clusters (e.g. ***Parasagitta spp.***^**1**^).

The hierarchical cluster analysis showed three environmental clusters among sample dates (Fig. 5A). The PCA showed that Cluster 1 contained mostly summer and early autumn months (July–October), characterized with high temperature, high biomass of small copepods, high nauplii abundance, low salinity and intermediate chlorophyll *a* concentration (Fig. 5B). Within Cluster 2, mostly autumn, winter and spring months were captured (November–June), characterized by intermediate to low temperature, low prey variables and chlorophyll *a* concentration, and intermediate to high salinity. Cluster 3 comprised of months during the spring bloom (April–May) comparable to Cluster 2, but with high chlorophyll *a* concentration and high *Calanus* spp. (small & large) biomass.

Beta diversity (*i.e.*, Bray–Curtis dissimilarities of fourth-root transformed abundance data) of GZP among these environmental clusters was assessed with a NMDS showing a relatively high stress value of 0.201 (Supplementary Fig. 4). Significant differences in beta diversity were found among all environmental clusters (PERMANOVA, R2 = 0.084, *F* = 5.74, *p* = 0.001; MANOVA, *p *_*cluters 1–2, 1–3,* & 2–3_ = 0.003), with the assumption of homogeneous dispersion being met (ANOVA, *F* = 1.96, *p* = 0.15). The relationship between taxa and the environmental clusters was visualized with a CAP (Fig. 5C). Indicator species analysis found four taxa to be indicative of the warmer, small copepod- and nauplii-rich Cluster 1: the Appendicularia genus *Oikopleura* spp. (*r. g* = 0.388, *p* = 0.001), the Chaetognatha genus *Parasagitta* spp. (*r. g* = 0.318, *p* = 0.020), the Cnidaria species *A. digitale* (*r. g* = 0.323, *p* = 0.017), and undetermined Hydromedusae (*r. g* = 0.359, *p* = 0.013). In addition, Chaetognatha undet. showed a weaker but significant association with the combined Cluster 1 + 3 group (r.g = 0.29, p = 0.044), which stretches across the productive season with high temperature, chlorophyll *a* concentration, copepod biomass and low salinity. No taxa were uniquely associated with Cluster 2 or Cluster 3 alone. *Mertensia* spp. was excluded from the multivariate analyses because no abundance data were available for dates where complete environmental data existed.

### Multiannual trends, interannual variability, and exceptional mass abundances

**Fig. 6:**
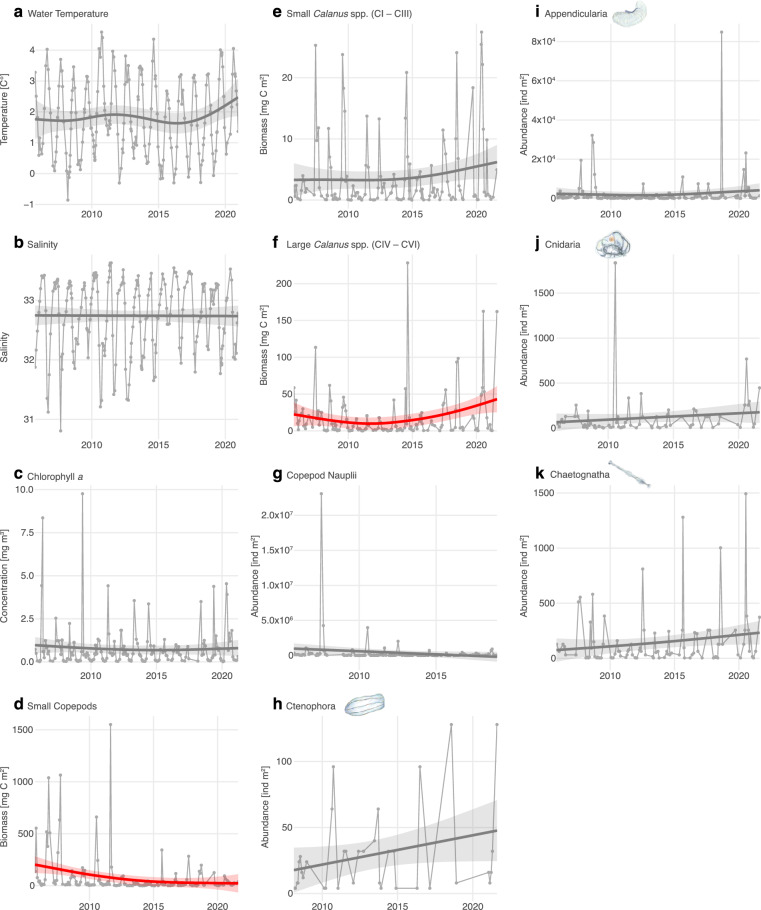
Multiannual trends of temperature (**a**), salinity (**b**), chlorophyll *a* (**c**), small copepod biomass (**d**), small *Calanus* spp. biomass (**e**), large *Calanus* spp. biomass (**f**), nauplii abundance (**g**), Ctenophora abundance (**h**), Appendicularia abundance (**i**), Cnidaria abundance (**j**), and Chaetognatha abundance (**k**) from October 2005–August 2021 at the outer fjord of Nuup Kangerlua. Trends were observed with generalized additive models (GAMs). Significant trends were indicated by fitted lines showing GAM predictions and 95% confidence intervals (shaded areas) in red and insignificant trends in grey.

Generalized additive models (GAMs) revealed distinct long-term trajectories across the modeled variables (Fig. 6; Supplementary Table 5). Significant non-linear trends were observed for small copepod biomass (edf = 1.95; *p* = 0.0029; *D*^*2*^ = 0.09) and large *Calanus* spp. biomass (edf = 2.43; *p* = 0.0142; D^2^ = 0.09), both characterized by substantial interannual fluctuations. In contrast, the environmental variables and the four GZP groups did not exhibit significant long-term trends, obscured by high interannual variability and stochastic “bloom” events.

Despite the lack of long-term trends in GZP, several exceptional mass abundance events were recorded. Ctenophora showed peaks in density (96 ind m^−2^) on 27 September 2010 and 27 June 2016, with the highest peak (128 ind m^−2^) recorded on 30 July 2018 and 9 August 2021. Appendicularia displayed the highest interannual variability, with highest densities observed of on 22 August 2008 (28,550 ind m^−2^), 24 August 2018 (84,864 ind m⁻2), and 16 July 2020 (23,211 ind m^−2^). Cnidaria showed maximum abundances on 20 July 2010 (1,835 ind m^−2^), 10 August 2020 (768 ind m^−2^), and 9 August 2021 (448 ind m^−2^). Chaetognatha exhibited peaks on 27 August 2015 (1,280 ind m^−2^), 30 July 2018 (1,003 ind m^−2^), and 16 July 2020 (1,493 ind m^−2^). As these findings are based on exploratory analyses of data with limited reliability, results should be interpreted with caution.

## Discussion

### GZP seasonal abundances and environmental drivers

Long-term year-round sampling is crucial for understanding the patterns of ecosystem dynamics and their responsible drivers for assessments and predictions of ecosystem changes under climate change. Yet, year-round multiyear GZP monitoring programs integrating abiotic and biotic drivers are rare. Using a 16-year time series, we show that prey availability is a central driver of predatory GZP (*i.e.*, Chaetognatha) seasonality, often being equal to or exceeding the correlation with temperature. The particle-feeding Appendicularia showed a similar pattern, yet the correlation with copepod prey variables might rather be due to co-occurrence or of indirect nature (e.g., increased particle accumulation) than reflecting a direct trophic link. This suggests that most of the present GZP assemblage can quickly exploit warmer, food-rich conditions during summer for growth and reproduction, likely due to the presence of boreo-Arctic species instead of true cold water adapted species like *Sminthea arctica*^30,60^. Comparisons to other high-latitude systems indicate that the seasonal succession observed in Nuup Kangerlua, characterized by increasing GZP abundances in spring and peak densities in summer, generally aligns with patterns reported across Arctic^29,61–63^ and sub-Arctic^57,59,64,65^ regions influenced by Atlantic water masses. Despite regional differences in hydrography, sampling depth, and species composition, a consistent pattern emerges: GZP abundance tends to peak during warm, food-rich summer periods, with timing shaped by local phenology and water-mass structure, typically between June and September.

For Ctenophora, the dominance of *Beroe* spp. and comparatively lower contributions of *Mertensia ovum* and *Bolinopsis infundibulum* are consistent with observations from Norwegian and Svalbard fjords, though seasonal peaks occurred slightly later than in some coastal systems^59,62–64,66^. Ctenophora abundances correlated the strongest positively with small *Calanus* spp. biomass, and slightly less with large *Calanus* spp. biomass, nauplii abundance and temperature, while salinity was not a significantly correlating variable, the latter in contrast to the other groups. Especially for the genus *Beroe*, several studies have demonstrated the lower importance of salinity in the Fram Strait and in the European Arctic^30,60,67^. Further, the positive correlation with the chlorophyll *a* concentration was stronger than for the other three groups. The seasonal abundance pattern coincided with the beginning of the annual phytoplankton spring bloom and abundances seemed to remain high as long as the chlorophyll *a* concentration did^68^. Ctenophora are not known to feed on phytoplankton, however, common tentacularian species like *Mertensia ovum*, feed on herbivorous zooplankton^33,69^. While Cirripedia and Bivalvia larvae highly dominate the zooplankton ensemble in May, the biomass of the herbivorous *Calanus* spp., which correlated strongly with Ctenophora abundances, usually peaked in June in the surface layer and showed a strong positive correlation to chlorophyll *a* with a one to two month lag^70^. Conversely, 57.2% of these Ctenophora were identified as *Beroe* spp., a genus known to exclusively feed on other Ctenophora and not on herbivorous mesozooplankton^71^. However, when the population of *M. ovum* feasts on the high copepod biomass^33^, *Beroe* spp. may decimate the high abundance of *M. ovum* and profit indirectly from high mesozooplankton biomass to potentially increase their own abundance. Evidence for this could be the low abundance contributions of *M. ovum*.

Appendicularia, the most abundant GZP group in Nuup Kangerlua, mainly *F. borealis* and *Oikopleura* spp., mirrored patterns observed in other high-latitude systems^61,62,72^. Appendicularia, the only non-predatory taxon among the four investigated groups, showed extreme boom-bust cycles. Their diet was described to consist mostly of phytoplankton, but also of various detritus particles that are caught in their mucus houses^73^. However, peak Appendicularia abundances spanning all taxa coincided later with the summer phytoplankton bloom, further associated with high biomass of small copepods and nauplii abundance. While it is hypothesized that Appendicularia abundance is negatively correlated with occurrence of other organisms utilizing the same food source, such as the small, omnivorous particle-feeding copepods *Oithona* spp., *Triconia* spp. or *Microsetella norvegica*, this can be ruled out here as their peak abundances co-occur^70,74^. Further, it is known that these copepods actually feed on discarded Appendicularia housings^45,75,76^. Appendicularia could possibly profit from the overall high copepod biomass that increases the amount of fecal particles and broken up phytoplankton shells at that time^70^, resulting in a positive feedback-loop. Full development within days and high reproduction could then be the reason for greatly increased abundances during favorable conditions^77^, e.g., irregular peak biomass events of copepods causing elevated particle accumulation. Interestingly, *Oikopleura* spp. abundances did not appear to be strongly linked to chlorophyll *a* directly and the annual spring bloom around May. Instead, they showed a strong negative correlation with salinity and were associated with the warm and intermediate chlorophyll *a* concentrations during the summer/autumn months. In contrast, *Fritillaria borealis* abundances were not influenced by salinity but showed a strong direct correlation with chlorophyll *a* concentrations. These observations agreed with pan-Arctic modelling approaches on *O. vanhoeffeni* and *F. borealis*^30^, indicating different ecological niches of the two genera as already suggested for tropical region congeners^78^ and differing feeding preferences on irregular phytoplankton or association with biomass peaks of small copepods.

Among Cnidaria, *A. digitale* was the most abundant taxon identified to species level, consistent with findings from Svalbard fjords^66^, Norwegian fjords^57,64,65^ and the Barent Sea^61,62^. However, while studies from lower latitudes reported hydrozoan peaks in June, abundances in our study reached their maximum in July, likely reflecting the later onset of the productive season at higher latitudes^57,64,65^. Cnidaria abundances were strongly associated with high water temperature and all prey availability variables on an equal level, low salinity and only moderately linked to chlorophyll *a* concentration. This weak direct correlation with chlorophyll *a* concentration and strong association with warm, copepod-rich periods might indicate that overall Cnidaria feed heavily on the copepod and nauplii assemblage throughout summer^70^. Contrastingly, the common *A. digitale* showed a strong single dependency on the spring-bloom-associated *Calanus* spp., even though diet analyses of this small Trachymedusae revealed diverse feeding on many different small copepods, other zooplankton and partly on phytoplankton^37,79^. Yet, both unidentified Hydromedusae and *A. digitale* were indicator taxa of the warm and prey-rich summer/autumn periods. Siphonophorae on the other hand seemed to be more dependent on the chlorophyll *a* concentration compared to other Cnidaria. This could be an indication of differing diets, and since siphonophores are assumingly carnivorous^80^, this could be explained by a stronger dependency on other non-copepod grazers of the spring bloom, for instance Cirripedia larvae.

The Chaetognatha community was comparable to other high latitude systems, with *Parasagitta* dominating summer assemblages, and high abundances of the deeper distributed *E. hamata*^29,62,63,81^. Lower contributions of deeper-dwelling taxa in comparison to other studies likely reflect vertical distribution patterns relative to the maximum sampling depth^58,63,72^. Further, the common large chaetognath *Pseudosagitta maxima* was completely missing in this monitoring, likely due to sampling limitations regarding the collection of larger taxa. Overall, Chaetognatha also increased their abundances in summer, together with rising temperature and very pronouncedly with prey availability. While some Chaetognatha species, for example *E. hamata*, have been considered to feed partly on phytoplankton^82^, this trophic relationship can be neglected here, since most of the sampled chaetognaths were identified as the carnivorous *Parasagitta* spp. In addition, our observations highlight their primarily carnivorous dietary preference^54,83^ and association with warming conditions in the surface layer. Nevertheless, as chlorophyll *a* concentrations impact copepod phenology, it should not be neglected in the context of trophodynamics of Chaetognatha.

In summary, while temperature has often been considered the primary driver of GZP dynamics at high latitudes, our long-term data align with observations from other high-latitude systems and show that food availability is at least equally important, and in several cases more strongly associated with seasonal abundance patterns.

### Exceptional mass abundances and potential drivers

Generalized additive models could only identify multiannual trends for the biomass of small copepods and large *Calanus* spp. These trends are discussed in detail by Detsch et al. (in review). In summary, on average cold periods (below 1.9 °C; 2005–2009 & 2015–2019;^84^) were characterized by high or regenerating copepod populations, while the warm period (2010–2014) was characterized by low copepod biomass. For the four GZP groups, a similar pattern could not be identified, possibly due to the inconsistency in sampling events and high variability in the dataset, highlighting the need for specialized GZP monitoring efforts to capture these dynamics more holistically in the future. Nevertheless, exceptional mass occurrences were often associated with distinct hydrographic anomalies. Several events coincided with warm water conditions, particularly in 2020 and 2021, when exceptionally high upper water column temperatures occurred simultaneously with peak abundances of Ctenophora, Appendicularia, Cnidaria, and Chaetognatha^84,85^. These synchronous increases suggest a potential community-level response to warm phases, consistent with findings from the Norwegian and Barents Seas, where Cnidaria and Ctenophora abundances are positively correlated with sea surface temperature^29^. Certain water masses can also be associated drivers of GZP blooms in the Arctic. Recent work in the Norwegian Arctic reported that relatively warm, saline Atlantic-water masses host enhanced GZP abundances^66^. This was especially the case for the Ctenophora *B. infundibulum* in the Barents Sea and *Beroe* spp. in Krossfjorden^66^, indicating that the hydrographic structure and the composition of water masses is a key factor modulating GZP dynamics in the Arctic. Conversely, several cold-water events also coincided with high GZP densities. In 2008, exceptionally high Appendicularia abundances overlapped with periods of pronounced cooling and inflow of Baffin Bay Polar Water in 2008^84,86^. Similarly, high Ctenophora and Chaetognatha densities in 2015 and 2016 occurred during another Baffin Bay Polar Water inflow, suggesting that cold-adapted taxa may benefit directly from lower temperature or indirectly from altered prey availability and predator interactions during such periods. Glacier activity also appeared to influence zooplankton assemblages. During summer 2018, intense glacier calving and thinning coincided with high densities of Ctenophora, Appendicularia, and Chaetognatha, despite overall cold annual temperatures^84^. Sub-glacial discharge and local meltwater input from icebergs could potentially have increased the nutrient availability through upwelling fueling primary production^87^. Thus, elevated chlorophyll *a* concentrations in 2018^85^ may have further contributed to enhanced productivity and secondary production. Overall, these observations suggest that GZP blooms in Nuup Kangerlua are not solely associated with warmer water conditions^25,29,31,32^. All exceptional densities, however, were recorded during summer, coinciding with peak copepod biomass (Detsch et al. in review). Although Appendicularia do not feed directly on copepods, their peak densities in 2007, 2008, and 2018 coincided with high biomass of *Metridia*, *Oithona*, *Triconia*, and *Calanus* species (Detsch et al. in review), suggesting that enhanced particle accumulation linked to copepod production may indirectly support Appendicularia feeding^73^. Similarly, peaks of Ctenophora, Cnidaria, and Chaetognatha, in 2016 and 2018 overlapped with copepod maxima, reflecting their role as copepod predators (Detsch et al. in review).

### Potential limitations of this study

As with most long-term plankton time series, interpretation of the results should consider constraints related to sampling methodology, spatial coverage, and taxonomic resolution. The use of a fine-mesh net (45 µm) has two main constraints: it effectively captures small mesozooplankton, but underestimates larger zooplankton taxa^88,89^ and sampling efficiency may be further reduced during periods of high phytoplankton biomass due to net clogging. However, because sampling methodology remained consistent throughout the study period, such biases are expected to be systematic and relative seasonal patterns and temporal trends remain consequently robust. The sampling location at the outer fjord (GF3) may not be representative of the entire fjord system as already demonstrated for the copepod community^72,90^. In addition, sampling was restricted to the upper 100 m, potentially underrepresenting taxa with deeper vertical distributions or pronounced vertical migrations. Seasonal vertical and horizontal migrations documented for several gelatinous taxa suggest that low winter abundances at the outer fjord may reflect redistribution or advection rather than mortality, particularly given strong tidal mixing in this region^59,91^. Taxonomic resolution represents an additional source of uncertainty, particularly for Cnidaria, where three quarters of the samples were classified as unidentified hydromedusae, limiting insights of species-specific responses to environmental change^30^. These limitations could be tackled by integration of recent methodological approaches such as environmental DNA analysis of seawater, which have the benefit of providing a higher taxonomic resolution^52^ as well as optical surveys and benthic sampling for documenting different life stages not limited to the adult medusa stage^53^. Consequently, future assessments integrating a more comprehensive methodological toolkit will be critical for detecting potential shifts in GZP community composition. Despite these limitations, the consistency of sampling year-round over 16 years provides a robust basis for identifying seasonal relationships between GZP and environmental drivers, particularly food availability.

## Conclusions

Long-term, year-round monitoring is crucial to understand ecological dynamics in Arctic-latitude marine systems, where strong seasonal abundance patterns and rapid environmental change interact to shape biological communities. Our findings on GZP distribution demonstrate that such sustained observations are required to move beyond frameworks only focusing on abiotic variables and to explicitly account for trophic structure and phenology when assessing and projecting GZP dynamics.

By resolving seasonal cycles across multiple trophic levels over 16 years, this study shows that the responses of gelatinous taxa to warming are closely linked to trophic conditions, a pattern that appears consistent across Arctic and sub-Arctic high-latitude systems. This underlines the importance of sustained time series programs for establishing ecological baselines and quantifying natural variability in Arctic marine ecosystems. As ongoing warming, freshwater input, and changes in hydrography continue to alter ecosystem dynamics, the long-term perspective provided here will be crucial for detecting future changes in pelagic community structure and dynamics.

## Methods

### Study area and zooplankton sampling

**Fig. 7:**
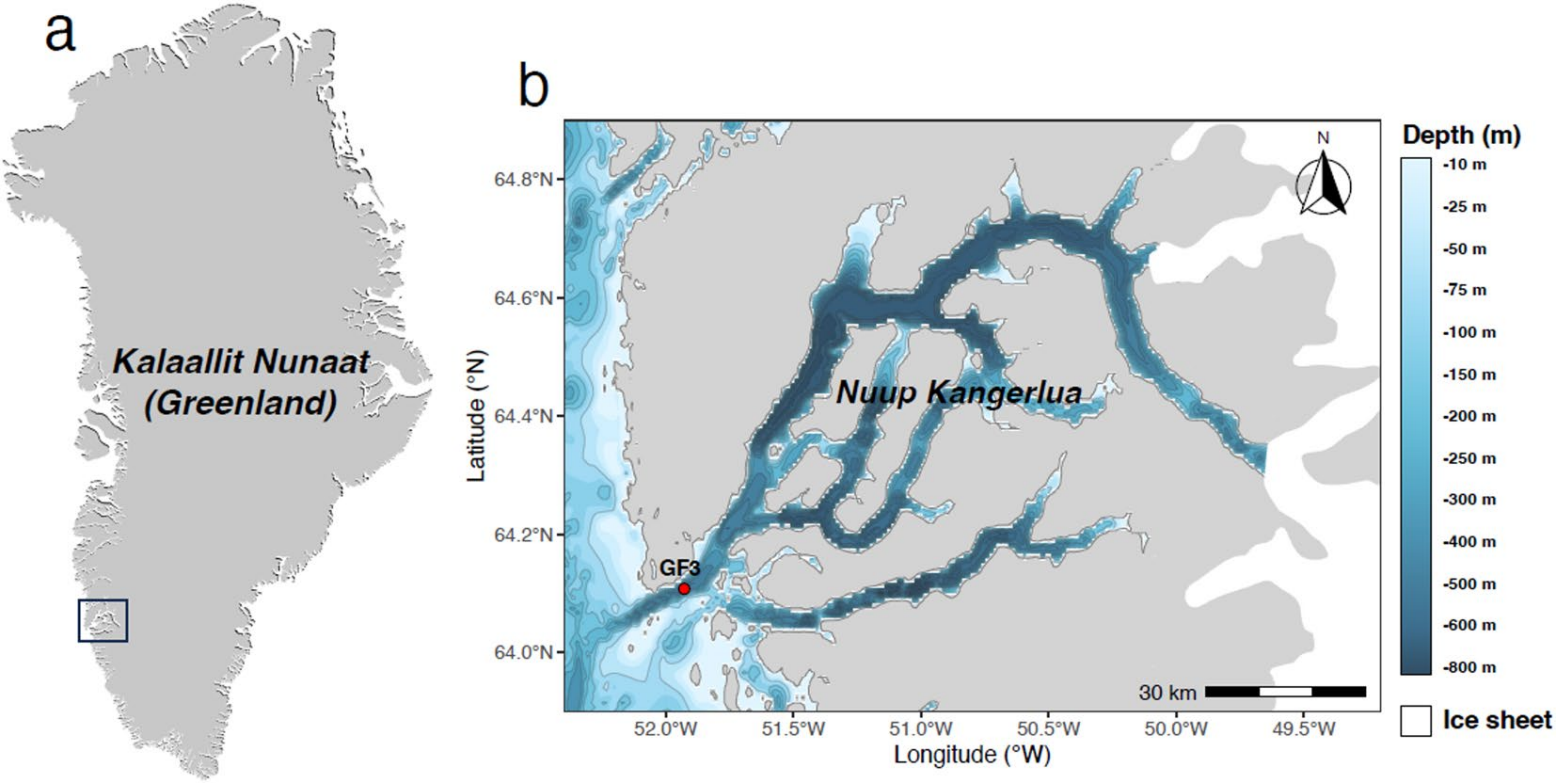
Overview of the study area in Greenland (**a**) and the Nuup Kangerlua fjord system, with location of the sampling site GF3 (**b**).

The fjord system Nuup Kangerlua (formerly Godhåbsfjord) is located approximately 300 km south of the Arctic Circle in Southwest Greenland, and is one of the largest fjord systems in Greenland, with a total surface area of 2013 km^2^ and an average depth of ~ 250 m^92^. MarineBasisNuuk (MBN), a subprogram of the Greenland Ecosystem Monitoring (GEM) program (g-e-m.dk), has established a long-term zooplankton monitoring at the outer fjord region. From October 2005 to August 2021, a total of 154 monthly sampling events were conducted (up to 12 per year) to infer hydrographical parameters, phytoplankton, and zooplankton community composition at the main station GF3 (64°07’N, 51°53’W). Station GF3 is located in the outer sill region of the fjord and has a depth of ~ 350 m (Fig. 7). As part of the MBN (GEM), zooplankton samples were collected with triplicate net hauls of a WP2 net with a mesh size of 45 µm, from 100 to 0 m. In January 2019, the WP2 net was lost, resulting in an eight-month gap until August 2019. After this gap, there was no copepod nauplii abundance data available yet. Zooplankton samples were preserved in borax-buffered formaldehyde (4% final concentration) and morphologically identified to the lowest taxonomic level possible. At the Arctic Agency, Poland, zooplankton specimens were enumerated from a volumetric subsample and prosome lengths of copepods (CI–CVI) were measured of 10 randomly selected individuals of each respective lowest possible copepod taxon, to calculate their respective biomass according to Arendt et al.^70^. Taxonomic names used are as currently accepted in the World Register of Marine Species. Detailed information on the general mesozooplankton and copepod community has been provided earlier.

### Environmental variables

Each month, vertical profiles of temperature and salinity were obtained using a CTD profiler (Seabird, SBE 19plus). Water samples were collected at depths of 1, 5, 10, 15, 20, 30, 50 and 100 m using a 5L Niskin water sampler for chlorophyll *a* measurements. Hydrographic conditions, water chemistry, microplankton dynamics, phytoplankton community composition, biomass, and primary production data have been reported elsewhere^68,84,86,92–95^. The water temperature, salinity, and chlorophyll *a* concentration values per sampling date were averaged for the upper 100 m. Variables for prey availability were small copepod biomass (copepodite stages CI–CVI of *Acartia* spp., *Centropages* spp., *Metridia* spp., *Microcalanus* spp., *Microsetella norvegica*, *Oithona* spp., *Pseudocalanus* spp., *Temora* spp., and *Triconia* spp.), small *Calanus* spp. (CI–CIII) biomass, large *Calanus* spp. (CIV–CVI) biomass, and copepod nauplii abundance (all species). The distinction between prey types was done to account for phenological and size dependent differences within the heterogenic copepod community. The biomass was calculated from the prosome length as reported in earlier studies^70,72,90^.

## Statistical analyses

### Trophic coupling of the copepod community with chlorophyll *a* concentration

The influence of chlorophyll *a* on the copepod community (small copepod biomass, small *Calanus* spp. biomass, large *Calanus* spp. biomass and copepod nauplii abundance) was assessed, including potential delayed effects. All environmental and biological variables were regularized to a monthly frequency using the *regul()* function (package ‘pastecs’ v.1.4.2; *method* = *“linear”*) to account for uneven sampling intervals^96^. Lagged chlorophyll *a* variables (0–4 months) were generated, and Spearman rank correlations were computed between each copepod group and chlorophyll *a* at each lag.

### Assessment of GZP community composition and seasonal abundance patterns

From the triplicate net hauls the average abundance of each GZP taxon was calculated for every sampling date. To present the community composition, each record of a taxon was assigned to their respective group (Ctenophora, Appendicularia, Cnidaria, or Chaetognatha), summed up and their relative contribution was calculated. For the seasonal abundance pattern of the four groups, each sampling record was sorted by month and log-transformed for better visualization. To evaluate seasonal differences in Ctenophora, Appendicularia, Cnidaria, and Chaetognatha abundance, months were grouped into four seasons: winter (January–March), spring (April–June), summer (July–September), and autumn (October–December). As the residuals of the linear models met the assumptions of normality (Shapiro–Wilk test) and homogeneity of variance (Bartlett test), the differences in abundance among seasons were assessed using an ANOVA significance test, followed by pairwise Tukey Honestly Significant Difference (HSD) test using the False Discovery Rate (FDR) method^97^ adjustment for multiple comparisons in R v.4.1.3^98^. Differences were considered statistically significant at a significance level of α = 0.05.

### Correlations between GZP abundances and environmental or trophic drivers

Spearman rank correlations were used to explore pairwise associations between GZP abundances (on phylum-level and lowest possible taxonomical level) and the seven environmental variables temperature, salinity, chlorophyll *a* concentration, small copepod biomass, small *Calanus* spp. biomass, large *Calanus* spp. biomass, and copepod nauplii abundance of the upper 100 m. Correlations were computed on non-log transformed abundance data using pairwise deletion of missing values. To control for multiple comparisons, we adjusted *p*-values using the FDR method. Taxa with fewer than ten observations with corresponding environmental parameter records were excluded. All plots were created in R Studio with ‘ggplot2’ v.3.5.1^99^ and edited in Affinity Publisher 2 v.2.6.5.

Environmental clustering and associated GZP community structureMultivariate analyses were performed to describe the environmental conditions at the sampling dates and the composition of associated GZP communities. Relationships among environmental variables were first explored with pairwise draftsman plots, created using the package ‘GGally’ v.2.2.1^100^. Inspection of these plots indicated that chlorophyll *a* concentration, small copepod biomass, small *Calanus* spp. biomass, large *Calanus* spp. biomass and copepod nauplii abundance required log-transformation [log(X + 1)] (Supplementary Figs. 2 and 3). All environmental variables were then standardized using the *scale()* function before further analyses. Prior to correlation analysis, multicollinearity among the environmental variables was assessed using variance inflation factors (VIF). Temperature, salinity, chlorophyll *a*, small copepod biomass, small *Calanus* spp. biomass, large *Calanus* spp. biomass and copepod nauplii abundance all showed acceptable VIF values (1.41–3.19), indicating no problematic collinearity among predictors. Environmental groupings were identified through hierarchical clustering based on Euclidean distances and Ward’s linkage method. A Principal Component Analysis (PCA) was used to visualize the combined variance explained by the environmental variables and to illustrate the separation among environmental clusters.

Beta diversity of GZP among samples was calculated using Bray–Curtis dissimilarities of fourth-root transformed abundance data. Variation in community composition among environmental clusters was visualized with Non-metric Multidimensional Scaling (NMDS). Overall and pairwise differences were assessed with a Permutational Multivariate Analysis of Variance (PERMANOVA) using 999 permutations (function *adonis2*, package ‘vegan’ v.2.7.1)^101^ and permutational MANOVA (function *pairwise.perm.manova*, package ‘RVAideMemoire’ v.0.9.83.12)^102^, and FDR corrected *p*-values. To relate species composition to environmental patterns, a Canonical Analysis of Principal Coordinates (CAP) was performed, and species vectors were fitted onto the ordination. Finally, indicator taxa for each environmental cluster were identified using indicator species analysis on untransformed abundance data (package ‘indicspecies’ v.1.8)^103^, applying the point-biserial correlation coefficient (*r.g*) as the association statistic and testing significance by permutation. *Mertensia* spp. was excluded from the multivariate analyses because no abundance data were available for dates where complete environmental data existed. All plots were created in R Studio with ‘ggplot2’.

Multiannual trends in environmental variables and zooplankton communities To assess multiannual trends in environmental variables (temperature, salinity, and chlorophyll *a*) and zooplankton (small copepod biomass, small *Calanus* spp. biomass, large *Calanus* spp. biomass, nauplii abundance, Ctenophora abundance, Appendicularia abundance, Cnidaria abundance, and Chaetognatha abundance) from October 2005 to August 2021 in the upper 100 m at the outer fjord of Nuup Kangerlua, generalized additive models (GAMs) were applied. Long-term trends were analyzed on untransformed raw data using the *gam()* function from the R package ‘mgcv’ v.1.9–3^104^. Temporal trends were modeled as smooth functions of time using thin-plate regression splines with a basis dimension of *k* = 10. Smoothing parameters were estimated via restricted maximum likelihood (REML). Trends were considered statistically significant at α = 0.05 based on the smooth term, and model fit was assessed using the proportion of deviance explained (*D*^*2*^). Model diagnostics were performed using the *gam.check* function to ensure basis dimension adequacy and residual normality. All analyses were conducted in RStudio, and plots were produced using ggplot2.

## Supplementary Information

Supplementary Information.

## Data Availability

The data that supports the findings of this study is available on g-e-m.dk under 10.17897/VE1B-1706. For further information, please contact the corresponding authors.

## Code availability

The computer code to perform statistical analyses is accessible on Zenodo (10.5281/zenodo.20744093).
