# Supplementary material for: 16 years of gelatinous zooplankton seasonal abundances reveal food availability and temperature as key drivers in Arctic latitudes
Source: Sci Rep. 2026 Jun 30;16:19912. doi: 10.1038/s41598-026-60064-1 (PMC13320164; doi:10.1038/s41598-026-60064-1)
Supplement: Supplementary file 1 — Supplementary Information. [file 41598_2026_60064_MOESM1_ESM.docx]

**Supplementary Material:** **16 years of gelatinous zooplankton seasonal abundances reveal food availability and temperature as key drivers in Arctic latitudes**

**Christian W. G. Detsch^1, 2, 3 *^, Thomas Juul-Pedersen^3 *^, Gerlien Verhaegen^4^, Charlotte Havermans^1, 2^, Doreen Kohlbach^2^**

^1^ University of Bremen, James-Watt-Straße 1, 28359 Bremen, Germany

^2^ Alfred Wegener Institute Helmholtz-Center for Polar and Marine Research, Am Handelshafen 12, 27570 Bremerhaven, Germany

^3^ Greenland Institute of Natural Resources, Kivioq 2, 3900 Nuuk, Greenland

^4^ Advanced Institute for Marine Ecosystem Change (WPI-AIMEC), Japan Agency for Marine-Earth Science and Technology, 3173-25 Showa-cho, Kanazawa-ku, Yokohama-shi, Kanagawa 236-0001, Japan

*** Correspondence:**Christian W. G. Detsch
detsch-christian@t-online.de

Thomas Juul-Pedersen

thpe@natur.gl

# Supplementary Figures and Tables

## Supplementary Figures

**Supplementary Figure 1.** Lagged correlations between chlorophyll-a concentration and prey abundance. Spearman correlation coefficients (ρ) are shown for lags of 0–4 months between monthly-regularized chlorophyll *a* concentration and four prey variables (small copepods biomass, small *Calanus* spp. (CI–CIII) biomass, large *Calanus* spp. (CIV–VI) biomass, and nauplii abundance). Positive values indicate higher prey availability following higher chlorophyll *a* concentrations at the specified lag, whereas negative values indicate inverse relationships. The dashed horizontal line marks zero correlation.

**Supplementary Figure 2.** Draftsman Plot of the four environmental variables water temperature, salinity, chlorophyll *a* concentration, small copepods biomass, small *Calanus* spp. (CI–CIII) biomass, large *Calanus* spp. (CIV–VI) biomass, and copepod nauplii abundance, without data transformation.

**Supplementary Figure 3.** Draftsman Plot of the four environmental variables water temperature, salinity, log-transformed +1 chlorophyll a concentration, log-transformed +1 small copepods biomass, log-transformed +1 small Calanus spp. (CI–CIII) biomass, log-transformed +1 large Calanus spp. (CIV–VI) biomass, and log-transformed +1 copepod nauplii abundance.

**Supplementary Figure 4.** NMDS ordination of gelatinous zooplankton community composition based on Bray–Curtis dissimilarities of fourth-root transformed abundance data. Points represent individual sampling events, colored according to environmental clusters identified from hierarchical clustering of environmental variables. Ellipses indicate 95% confidence intervals around the centroids of each cluster.

## Supplementary Tables

**Supplementary Table 1.** Results of Spearman rank correlation analyses between the four copepod variables (small copepod biomass, small *Calanus* spp. (CI–CIII) biomass, big *Calanus* spp. (CIV–CVI) biomass and copepod nauplii abundance) and the 0–4 months lagged chlorophyll *a* concentration. Exact correlations coefficients should be handled with care as they are based on regularized data.

| Variable | Lag | Rho | p-value |
| --- | --- | --- | --- |
| SMALL_COPEPODS | 0 | 0.3419 | <0.0001 |
| SMALL_COPEPODS | 1 | 0.4336 | <0.0001 |
| SMALL_COPEPODS | 2 | 0.4406 | <0.0001 |
| SMALL_COPEPODS | 3 | 0.3616 | <0.0001 |
| SMALL_COPEPODS | 4 | 0.2056 | 0.0048 |
| SMALL_CALANUS | 0 | 0.4828 | <0.0001 |
| SMALL_CALANUS | 1 | 0.574 | <0.0001 |
| SMALL_CALANUS | 2 | 0.4659 | <0.0001 |
| SMALL_CALANUS | 3 | 0.1828 | 0.0123 |
| SMALL_CALANUS | 4 | -0.1351 | 0.0653 |
| BIG_CALANUS | 0 | 0.432 | <0.0001 |
| BIG_CALANUS | 1 | 0.5491 | <0.0001 |
| BIG_CALANUS | 2 | 0.4859 | <0.0001 |
| BIG_CALANUS | 3 | 0.2526 | 0.0005 |
| BIG_CALANUS | 4 | -0.0614 | 0.4031 |
| NAUPLII | 0 | 0.433 | <0.0001 |
| NAUPLII | 1 | 0.4787 | <0.0001 |
| NAUPLII | 2 | 0.4474 | <0.0001 |
| NAUPLII | 3 | 0.2925 | 0.0002 |
| NAUPLII | 4 | 0.0698 | 0.3879 |

**Supplementary Table 2.** Overview of all identified gelatinous zooplankton taxa, their total abundances [ind m^-2^] and their contribution to the abundance of their respective phylum.

| GZP Group | Taxon | Total Abundance [ind m^-2^] | Percentage |
| --- | --- | --- | --- |
| Ctenophora | *Beroe* spp. | 606 | 57.2% |
|  | Ctenophora undet. | 412 | 38.9 % |
|  | *Mertensia* spp. | 42 | 3.96 % |
| Chaetognatha | *Parasagitta* spp. | 5747 | 59.9 % |
|  | Chaetognatha undet. | 5496 | 39.1 % |
|  | *Eukrohnia* spp. | 144 | 1.02 % |
| Appendicularia | *Fritillaria borealis* | 175489 | 50.3 % |
|  | *Fritillaria* spp. | 108852 | 31.2 % |
|  | *Oikopleura* spp. | 57663 | 16.5 % |
|  | Appendicularia undet. | 6641 | 1.9 % |
| Cnidaria | Hydromedusae undet. | 6383 | 74.5 % |
|  | *Aglantha digitale* | 1228 | 14.3 % |
|  | *Muggiaea* spp. | 620 | 7.24 % |
|  | Siphonophorae undet. | 168 | 1.96 % |
|  | *Dimophyes* spp. | 168 | 1.96 % |

**Supplementary Table 3.** Results of ANOVA and pairwise Tukey HSD tests (Benjamini–Hochberg correction) assessing seasonal differences in abundance (or log-transformed abundance) of the four gelatinous zooplankton groups. Significant differences (*p* < 0.05) are highlighted in bold.

| Taxon | F (df) | p-value | Seasonal differences (Tukey HSD, adjusted) |
| --- | --- | --- | --- |
| Ctenophora | 11.38 (3,32) | <0.0001 | Spring–Fall (0.228); **Summer > Fall (0.0046)**; Winter–Fall (0.696); Summer–Spring (0.212); **Winter < Spring (0.0070)**; **Winter < Summer (<0.0001)** |
| Appendicularia | 30.92 (3,148) | <0.0001 | Spring–Fall (0.954); **Summer > Fall (<0.0001)**; **Winter < Fall (<0.0001)**; **Summer > Spring (<0.0001)**; **Winter < Spring (1.5×10⁻⁴)**; **Winter < Summer (<0.0001)** |
| Cnidaria | 14.96 (3,71) | <0.0001 | Spring–Fall (0.560); **Summer > Fall (0.00033)**; Winter–Fall (0.935); **Summer > Spring (0.0013)**; Winter–Spring (0.0978); **Winter < Summer (<0.0001)** |
| Chaetognatha | 39.45 (3,93) | <0.0001 | Spring–Fall (0.221); **Summer > Fall (<0.0001)**; **Winter < Fall (0.0245)**; **Summer > Spring (<0.0001)**; **Winter < Spring (<0.0001)**; **Winter < Summer (<0.0001)** |

**Supplementary Table 4.** Results of Spearman rank correlation analyses between the abundance of gelatinous zooplankton taxa and four environmental parameters (temperature, salinity, chlorophyll *a* concentration, and copepod biomass). Shown are sample sizes (*n*), Spearman correlation coefficients (ρ), unadjusted and Benjamini–Hochberg–adjusted *p*-values (*p* and *p*_adj), and corresponding significance levels (* = *p* < 0.05; ** = *p* < 0.01; *** = *p* < 0.001).

| **Taxon** | **Environmental variable** | **n** | **rho** | ***p*-value (unadjusted)** | ***p*-value (adjusted)** | **sig.** |
| --- | --- | --- | --- | --- | --- | --- |
| **Appendicularia undet.** | TEMPERATURE | 57 | 0.5508 | <0.0001 | <0.0001 | *** |
| ***Fritillaria borealis*** | TEMPERATURE | 92 | 0.5843 | <0.0001 | <0.0001 | *** |
| ***Fritillaria* spp.** | TEMPERATURE | 32 | 0.4302 | 0.014 | 0.0205 | * |
| ***Oikopleura* spp.** | TEMPERATURE | 108 | 0.5454 | <0.0001 | <0.0001 | *** |
| **Chaetognatha undet.** | TEMPERATURE | 38 | 0.4801 | 0.0023 | 0.0042 | ** |
| ***Eukhronia* spp.** | TEMPERATURE | 2 | N.A. | N.A. | N.A. |  |
| ***Parasagitta* spp.** | TEMPERATURE | 62 | 0.7379 | <0.0001 | <0.0001 | *** |
| ***Aglantha digitale*** | TEMPERATURE | 14 | 0.4928 | 0.0734 | 0.0936 |  |
| ***Dimophyes* spp.** | TEMPERATURE | 15 | 0.5615 | 0.0294 | 0.0399 | * |
| **Hydromedusae** | TEMPERATURE | 44 | 0.6306 | <0.0001 | <0.0001 | *** |
| ***Muggiaea* spp.** | TEMPERATURE | 16 | 0.6994 | 0.0026 | 0.0043 | ** |
| **Siphonophora undet.** | TEMPERATURE | 7 | 0.3349 | 0.4627 | 0.4986 |  |
| ***Beroe* spp.** | TEMPERATURE | 16 | 0.7263 | 0.0014 | 0.0028 | ** |
| **Ctenophora undet.** | TEMPERATURE | 14 | 0.6912 | 0.0062 | 0.01 | * |
| ***Mertensia* sp.** | TEMPERATURE | 3 | -0.5 | 0.6667 | 0.6877 |  |
| **TotalCtenophora** | TEMPERATURE | 27 | 0.7013 | <0.0001 | 0.0001 | *** |
| **TotalCnidaria** | TEMPERATURE | 63 | 0.6013 | <0.0001 | <0.0001 | *** |
| **TotalAppendicularia** | TEMPERATURE | 135 | 0.5601 | <0.0001 | <0.0001 | *** |
| **TotalChaetognatha** | TEMPERATURE | 84 | 0.6334 | <0.0001 | <0.0001 | *** |
| **Appendicularia undet.** | SALINITY | 57 | -0.0239 | 0.8599 | 0.8669 |  |
| ***Fritillaria borealis*** | SALINITY | 92 | -0.1449 | 0.1682 | 0.2002 |  |
| ***Fritillaria* spp.** | SALINITY | 32 | -0.4093 | 0.02 | 0.0278 | * |
| ***Oikopleura* spp.** | SALINITY | 108 | -0.4475 | <0.0001 | <0.0001 | *** |
| **Chaetognatha undet.** | SALINITY | 38 | -0.1964 | 0.2372 | 0.2696 |  |
| ***Eukhronia* spp.** | SALINITY | 2 | N.A. | N.A. | N.A. |  |
| ***Parasagitta* spp.** | SALINITY | 62 | -0.4632 | 0.0002 | 0.0003 | *** |
| ***Aglantha digitale*** | SALINITY | 14 | -0.3456 | 0.2261 | 0.2593 |  |
| ***Dimophyes* spp.** | SALINITY | 15 | -0.2631 | 0.3433 | 0.3798 |  |
| **Hydromedusae** | SALINITY | 44 | -0.3867 | 0.0095 | 0.0145 | * |
| ***Muggiaea* spp.** | SALINITY | 16 | -0.4142 | 0.1108 | 0.1371 |  |
| **Siphonophora undet.** | SALINITY | 7 | -0.197 | 0.672 | 0.6877 |  |
| ***Beroe* spp.** | SALINITY | 16 | -0.2023 | 0.4523 | 0.4917 |  |
| **Ctenophora undet.** | SALINITY | 14 | -0.3917 | 0.166 | 0.1996 |  |
| ***Mertensia* sp.** | SALINITY | 3 | 1 | <0.0001 | <0.0001 | *** |
| **TotalCtenophora** | SALINITY | 27 | -0.2966 | 0.133 | 0.163 |  |
| **TotalCnidaria** | SALINITY | 63 | -0.3759 | 0.0024 | 0.0043 | ** |
| **TotalAppendicularia** | SALINITY | 135 | -0.2981 | 0.0004 | 0.0009 | *** |
| **TotalChaetognatha** | SALINITY | 84 | -0.3068 | 0.0045 | 0.0075 | ** |
| **Appendicularia undet.** | CHLOROPHYLL | 45 | 0.5648 | 0.0001 | 0.0001 | *** |
| ***Fritillaria borealis*** | CHLOROPHYLL | 78 | 0.5667 | <0.0001 | <0.0001 | *** |
| ***Fritillaria* spp.** | CHLOROPHYLL | 31 | 0.3823 | 0.0338 | 0.0449 | * |
| ***Oikopleura* spp.** | CHLOROPHYLL | 93 | 0.177 | 0.0897 | 0.1133 |  |
| **Chaetognatha undet.** | CHLOROPHYLL | 28 | 0.0824 | 0.6767 | 0.6877 |  |
| ***Eukhronia* spp.** | CHLOROPHYLL | 2 | N.A. | N.A. | N.A. |  |
| ***Parasagitta* spp.** | CHLOROPHYLL | 55 | 0.4592 | 0.0004 | 0.0009 | *** |
| ***Aglantha digitale*** | CHLOROPHYLL | 11 | 0.1655 | 0.6267 | 0.6528 |  |
| ***Dimophyes* spp.** | CHLOROPHYLL | 15 | 0.593 | 0.0198 | 0.0278 | * |
| **Hydromedusae** | CHLOROPHYLL | 40 | 0.3525 | 0.0257 | 0.0353 | * |
| ***Muggiaea* spp.** | CHLOROPHYLL | 15 | 0.7465 | 0.0014 | 0.0027 | ** |
| **Siphonophora undet.** | CHLOROPHYLL | 8 | -0.5455 | 0.1619 | 0.1965 |  |
| ***Beroe* spp.** | CHLOROPHYLL | 16 | 0.6047 | 0.0131 | 0.0195 | * |
| **Ctenophora undet.** | CHLOROPHYLL | 15 | 0.8013 | 0.0003 | 0.0007 | *** |
| ***Mertensia* sp.** | CHLOROPHYLL | 4 | -0.8 | 0.2 | 0.2315 |  |
| **TotalCtenophora** | CHLOROPHYLL | 28 | 0.5685 | 0.0016 | 0.003 | ** |
| **TotalCnidaria** | CHLOROPHYLL | 57 | 0.3151 | 0.017 | 0.0241 | * |
| **TotalAppendicularia** | CHLOROPHYLL | 115 | 0.4271 | <0.0001 | <0.0001 | *** |
| **TotalChaetognatha** | CHLOROPHYLL | 72 | 0.3995 | 0.0005 | 0.001 | ** |
| **Appendicularia undet.** | SMALL_COPEPODS | 62 | 0.6943 | <0.0001 | <0.0001 | *** |
| ***Fritillaria borealis*** | SMALL_COPEPODS | 103 | 0.6312 | <0.0001 | <0.0001 | *** |
| ***Fritillaria* spp.** | SMALL_COPEPODS | 37 | 0.4544 | 0.0047 | 0.0078 | ** |
| ***Oikopleura* spp.** | SMALL_COPEPODS | 124 | 0.7101 | <0.0001 | <0.0001 | *** |
| **Chaetognatha undet.** | SMALL_COPEPODS | 45 | 0.6672 | <0.0001 | <0.0001 | *** |
| ***Eukhronia* spp.** | SMALL_COPEPODS | 2 | N.A. | N.A. | N.A. |  |
| ***Parasagitta* spp.** | SMALL_COPEPODS | 69 | 0.7144 | <0.0001 | <0.0001 | *** |
| ***Aglantha digitale*** | SMALL_COPEPODS | 18 | 0.4353 | 0.071 | 0.0915 |  |
| ***Dimophyes* spp.** | SMALL_COPEPODS | 17 | 0.4617 | 0.0621 | 0.0809 |  |
| **Hydromedusae** | SMALL_COPEPODS | 53 | 0.5019 | 0.0001 | 0.0003 | *** |
| ***Muggiaea* spp.** | SMALL_COPEPODS | 19 | 0.7359 | 0.0003 | 0.0007 | *** |
| **Siphonophora undet.** | SMALL_COPEPODS | 9 | 0.2772 | 0.4701 | 0.5023 |  |
| ***Beroe* spp.** | SMALL_COPEPODS | 20 | 0.6456 | 0.0021 | 0.0039 | ** |
| **Ctenophora undet.** | SMALL_COPEPODS | 20 | 0.5753 | 0.008 | 0.0124 | * |
| ***Mertensia* sp.** | SMALL_COPEPODS | 4 | -0.8 | 0.2 | 0.2315 |  |
| **TotalCtenophora** | SMALL_COPEPODS | 36 | 0.6567 | <0.0001 | <0.0001 | *** |
| **TotalCnidaria** | SMALL_COPEPODS | 75 | 0.5406 | <0.0001 | <0.0001 | *** |
| **TotalAppendicularia** | SMALL_COPEPODS | 152 | 0.6961 | <0.0001 | <0.0001 | *** |
| **TotalChaetognatha** | SMALL_COPEPODS | 97 | 0.6555 | <0.0001 | <0.0001 | *** |
| **Appendicularia undet.** | SMALL_CALANUS | 57 | 0.7143 | <0.0001 | <0.0001 | *** |
| ***Fritillaria borealis*** | SMALL_CALANUS | 91 | 0.6241 | <0.0001 | <0.0001 | *** |
| ***Fritillaria* spp.** | SMALL_CALANUS | 32 | 0.2431 | 0.1801 | 0.2123 |  |
| ***Oikopleura* spp.** | SMALL_CALANUS | 108 | 0.4514 | <0.0001 | <0.0001 | *** |
| **Chaetognatha undet.** | SMALL_CALANUS | 41 | 0.463 | 0.0023 | 0.0042 | ** |
| ***Eukhronia* spp.** | SMALL_CALANUS | 2 | N.A. | N.A. | N.A. |  |
| ***Parasagitta* spp.** | SMALL_CALANUS | 64 | 0.6524 | <0.0001 | <0.0001 | *** |
| ***Aglantha digitale*** | SMALL_CALANUS | 17 | 0.574 | 0.016 | 0.023 | * |
| ***Dimophyes* spp.** | SMALL_CALANUS | 14 | 0.2468 | 0.3951 | 0.4332 |  |
| **Hydromedusae** | SMALL_CALANUS | 50 | 0.4175 | 0.0026 | 0.0043 | ** |
| ***Muggiaea* spp.** | SMALL_CALANUS | 14 | 0.7406 | 0.0024 | 0.0043 | ** |
| **Siphonophora undet.** | SMALL_CALANUS | 8 | -0.0273 | 0.9489 | 0.9489 |  |
| ***Beroe* spp.** | SMALL_CALANUS | 19 | 0.6024 | 0.0063 | 0.0102 | * |
| **Ctenophora undet.** | SMALL_CALANUS | 19 | 0.8676 | <0.0001 | <0.0001 | *** |
| ***Mertensia* sp.** | SMALL_CALANUS | 4 | 1 | <0.0001 | <0.0001 | *** |
| **TotalCtenophora** | SMALL_CALANUS | 34 | 0.7982 | <0.0001 | <0.0001 | *** |
| **TotalCnidaria** | SMALL_CALANUS | 69 | 0.5083 | <0.0001 | <0.0001 | *** |
| **TotalAppendicularia** | SMALL_CALANUS | 131 | 0.5314 | <0.0001 | <0.0001 | *** |
| **TotalChaetognatha** | SMALL_CALANUS | 89 | 0.664 | <0.0001 | <0.0001 | *** |
| **Appendicularia undet.** | BIG_CALANUS | 56 | 0.7273 | <0.0001 | <0.0001 | *** |
| ***Fritillaria borealis*** | BIG_CALANUS | 85 | 0.5864 | <0.0001 | <0.0001 | *** |
| ***Fritillaria* spp.** | BIG_CALANUS | 34 | 0.2922 | 0.0936 | 0.117 |  |
| ***Oikopleura* spp.** | BIG_CALANUS | 110 | 0.5196 | <0.0001 | <0.0001 | *** |
| **Chaetognatha undet.** | BIG_CALANUS | 41 | 0.5842 | 0.0001 | 0.0001 | *** |
| **Eukhronia sp.** | BIG_CALANUS | 2 | N.A. | N.A. | N.A. |  |
| **Parasagitta sp.** | BIG_CALANUS | 62 | 0.776 | <0.0001 | <0.0001 | *** |
| **Aglantha digitale** | BIG_CALANUS | 18 | 0.5846 | 0.0108 | 0.0163 | * |
| **Dimophyes sp.** | BIG_CALANUS | 15 | 0.1557 | 0.5796 | 0.614 |  |
| **Hydromedusae** | BIG_CALANUS | 48 | 0.566 | <0.0001 | 0.0001 | *** |
| **Muggiaea sp.** | BIG_CALANUS | 18 | 0.8175 | <0.0001 | 0.0001 | *** |
| **Siphonophora sp.** | BIG_CALANUS | 9 | 0.4258 | 0.2532 | 0.2852 |  |
| **Beroe sp.** | BIG_CALANUS | 20 | 0.6372 | 0.0025 | 0.0043 | ** |
| **Ctenophora undet.** | BIG_CALANUS | 18 | 0.5999 | 0.0085 | 0.0131 | * |
| **Mertensia sp.** | BIG_CALANUS | 4 | 0.4 | 0.6 | 0.6303 |  |
| **TotalCtenophora** | BIG_CALANUS | 34 | 0.7205 | <0.0001 | <0.0001 | *** |
| **TotalCnidaria** | BIG_CALANUS | 68 | 0.5631 | <0.0001 | <0.0001 | *** |
| **TotalAppendicularia** | BIG_CALANUS | 129 | 0.5207 | <0.0001 | <0.0001 | *** |
| **TotalChaetognatha** | BIG_CALANUS | 86 | 0.7509 | <0.0001 | <0.0001 | *** |
| **Appendicularia undet.** | NAUPLII | 46 | 0.851 | <0.0001 | <0.0001 | *** |
| **Fritillaria borealis** | NAUPLII | 86 | 0.7613 | <0.0001 | <0.0001 | *** |
| **Fritillaria sp.** | NAUPLII | 25 | 0.5271 | 0.0068 | 0.0107 | * |
| **Oikopleura sp.** | NAUPLII | 95 | 0.5715 | <0.0001 | <0.0001 | *** |
| **Chaetognatha undet.** | NAUPLII | 39 | 0.7147 | <0.0001 | <0.0001 | *** |
| **Eukhronia sp.** | NAUPLII | 1 | N.A. | N.A. | N.A. |  |
| **Parasagitta sp.** | NAUPLII | 45 | 0.8165 | <0.0001 | <0.0001 | *** |
| **Aglantha digitale** | NAUPLII | 12 | 0.6015 | 0.0385 | 0.0507 |  |
| **Dimophyes sp.** | NAUPLII | 14 | 0.3229 | 0.2601 | 0.2903 |  |
| **Hydromedusae** | NAUPLII | 41 | 0.6087 | <0.0001 | 0.0001 | *** |
| **Muggiaea sp.** | NAUPLII | 15 | 0.8384 | 0.0001 | 0.0002 | *** |
| **Siphonophora sp.** | NAUPLII | 3 | 1 | <0.0001 | <0.0001 | *** |
| **Beroe sp.** | NAUPLII | 11 | 0.6498 | 0.0304 | 0.0409 | * |
| **Ctenophora undet.** | NAUPLII | 16 | 0.5919 | 0.0157 | 0.0229 | * |
| **Mertensia sp.** | NAUPLII | 0 | N.A. | N.A. | N.A. |  |
| **TotalCtenophora** | NAUPLII | 23 | 0.7459 | <0.0001 | 0.0001 | *** |
| **TotalCnidaria** | NAUPLII | 58 | 0.6751 | <0.0001 | <0.0001 | *** |
| **TotalAppendicularia** | NAUPLII | 122 | 0.7242 | <0.0001 | <0.0001 | *** |
| **TotalChaetognatha** | NAUPLII | 71 | 0.734 | <0.0001 | <0.0001 | *** |

**Supplementary Table 5.** Summary statistics from generalized additive models (GAMs) evaluating multiannual trends (October 2005–August 2021) in environmental and zooplankton variables in the upper 100 m at the outer fjord of Nuup Kangerlua. Shown are the effective degrees of freedom (edf) of the smooth term, *p*-value, and proportion of deviance explained. Trends were considered statistically significant at α = 0.05.

| **Variable** | **EDF** | **p_value** | **Deviance_explained** |
| --- | --- | --- | --- |
| **Temperature** | 3.3583 | 0.1994 | 0.0418 |
| **Salinity** | 1.0003 | 0.945 | 0 |
| **Chlorophyll *a*** | 1.5297 | 0.5804 | 0.0101 |
| **Small_Copepods** | 1.946 | 0.0029 | 0.086 |
| **Small_Calanus** | 1.7301 | 0.2331 | 0.0301 |
| **Big_Calanus** | 2.4264 | 0.0142 | 0.0888 |
| **Nauplii** | 1.0012 | 0.0754 | 0.0251 |
| **Ctenophora** | 1.0002 | 0.0901 | 0.0822 |
| **Appendicularia** | 1.6914 | 0.4663 | 0.0163 |
| **Cnidaria** | 1.0007 | 0.2496 | 0.0182 |
| **Chaetognatha** | 1.1392 | 0.1026 | 0.0357 |
